# Tranexamic acid for the prevention of postpartum haemorrhage: the TAPPH-1 pilot randomized trial and lessons learned for trials in Canadian obstetrics

**DOI:** 10.1038/s41598-023-30947-8

**Published:** 2023-03-18

**Authors:** Asim Q. Alam, Jon Barrett, Jeannie Callum, Lilia Kaustov, Shelly Au, Andrew Fleet, Alex Kiss, Stephen Choi

**Affiliations:** 1 Department of Anesthesia, Sunnybrook Health Sciences Centre, University of Toronto, M3-200, 2075 Bayview Avenue, Toronto, ON M4N 3M5 Canada; 2 Department of Obstetrics and Gynaecology, Sunnybrook Health Sciences Centre, University of Toronto, Toronto, ON Canada; 3 Department of Pathology and Molecular Medicine, Queen’s University, Kingston, ON Canada; 4 Department of Research Design and Biostatistics, Sunnybrook Research Institute, Toronto, ON Canada; 5 Present Address: Department of Anesthesia, North York General Hospital, Toronto, ON Canada; 6 Present Address: Department of Obstetrics and Gynaecology, McMaster University, Hamilton, ON Canada

**Keywords:** Randomized controlled trials, Disease prevention

## Abstract

Postpartum haemorrhage (PPH) is a leading cause of maternal morbidity and mortality. While tranexamic acid (TXA) reduces bleeding and transfusion requirements in established PPH, we sought to determine the feasibility of conducting a fully powered trial assessing the effect of prophylactic tranexamic acid, prior to PPH onset, in a Canadian Obstetric setting. With institutional and Health Canada approval, consenting, eligible parturients (singleton, > 32 weeks gestation, vaginal or caesarian delivery) were randomly assigned to receive TXA (1 g intravenously) or placebo (0.9% saline) prior to delivery. Participants, investigators, data collectors/adjudicators, and analysis was blinded. The primary outcome was administration of study intervention to > 85% of randomized individuals. Secondary outcomes included recruitment rate (feasibility) and safety outcomes. Over 8 months, 611 were approached, 35 consented, and 27 randomized (14 TXA, 13 placebo). 89% of randomized participants received the assigned intervention. Recruitment fell below feasibility (23% target). No serious adverse outcomes occurred. Our pilot trial in a Canadian Obstetric setting was unable to demonstrate feasibility to conduct a large, multicentre trial to examine prophylactic use of tranexamic for PPH secondary to the complex regulatory requirements associated with a trial for an off-label, but commonly utilized intervention. These challenges should inform stakeholders on the resources and challenges of conducting future trials using off-label interventions.

Trial registration: www.clinicaltrials.gov, NCT03069859 (03/03/2017).

## Introduction

Postpartum haemorrhage (PPH) is a leading cause of maternal mortality in most locales worldwide and third highest in high human development index (HDI) countries^1^. Previously defined as blood loss greater than or equal to 500 mL following spontaneous vaginal delivery (SVD) or greater than or equal to 1000 mL following caesarean delivery (CD), the diagnosis of PPH is has been redefined to blood loss greater than 1000 ml or blood loss associated with signs or symptoms of hypovolemia within 24 h of delivery^2^. The incidence of PPH is 14.2% worldwide with regional variability (as high as 28% in Africa) and associated with 27.1% of maternal mortality^1,3^. However, the incidence of PPH in high HDI countries is rising, with a 22% relative increase in Canada (5.1–6.2%, from 2003 to 2010), and 26% in the United States (2.3–2.9%, from 1994 to 2006)^4,5^. Despite having among the most advanced healthcare systems in the world, PPH accounts for 11% of maternal deaths in the United States^6^. The morbidity associated with PPH is also increasing with higher rates of PPH associated transfusion and emergent hysterectomy^5,7^. One study estimated that direct hospitalization costs of a delivery in the United States increase from $5000 to $90,000 when PPH occurs, notwithstanding indirect costs of subsequent sequelae^8^. There is a compelling need for a safe, cost-effective preventative strategy to reduce PPH.

Tranexamic acid (TXA) is an anti-fibrinolytic compound that inhibits the conversion of plasminogen to plasmin, preventing fibrinolysis^9^. TXA has a proven safety profile and has level 1 evidence as well as being the standard of care to reduce blood loss, transfusion requirements, and/or mortality in multiple operative settings including cardiac, orthopedic, and trauma surgery. Notably, in all these settings, while standard of care, TXA is used off-label^10–12^. Indeed, the WOMAN trial demonstrated administering TXA, after PPH onset, reduces mortality^13^. However, because PPH is already established, significant morbidity and cost still exist. Prevention of PPH would be preferable and reduce morbidity further. Several meta-analyses conclude prophylactic TXA reduces the incidence of PPH, however all these expressed concerns regarding methodology of the various included trials and concluded that it was premature to recommend routine prophylactic use, instead calling for further investigation^14–16^. Because its use as an off-label therapy, any trials would require regulatory approval from the appropriate national agencies (e.g. FDA, Health Canada).

Prior to initiating a definitive trial, we aimed to conduct a pilot trial to determine the feasibility (recruitment potential, ability to adhere to study protocol) and identify potential logistical barriers. Our hypothesis for a definitive trial is that prophylactic administration of TXA (at time of shoulder delivery for spontaneous vaginal delivery or skin preparation for caesarian section) reduces the incidence of PPH associated with childbirth.

## Materials and methods

This pilot randomized trial was conducted at Sunnybrook Health Sciences Centre (SHSC), a tertiary care academic health sciences centre fully affiliated with the University of Toronto. SHSC is a high-risk obstetrical centre with approximately 4,000 deliveries annually. The study protocol was approved by the Research Ethics Board of SHSC (April 2017, REB ID# 418-2016) and registered with www.clinicaltrials.gov (03/03/2017, NCT03069859). Regulatory approval was sought and received from Health Canada (July 2017, Control# 207130) for off-label intravenous administration of TXA. Written informed consent was obtained from all participating subjects. Participants could withdraw at any point. Data collected until the time of withdrawal was retained. All research was conducted in accordance with relevant guidelines and regulations (Declaration of Helsinki, Good Clinical Practice, Tri-Couuncil Policy Statement Ethical Conduct for Research Involving Humans).

The protocol for the TAPPH-1 pilot study, designed in accordance with Standard Protocol Items: Recommendations for Interventional Trials (SPIRIT) principles, has been published previously^17^. The primary objective was to assess the feasibility of recruitment and administration of investigational product (IP) (TXA) in this population.

Patients eligible for inclusion were female ($\ge$ 18 years), with confirmed singleton pregnancies, gestational age > 32 weeks, via any delivery method (spontaneous vaginal delivery [SVD], elective/urgent caesarian delivery [CD]), irrespective of anesthetic plan (epidural, spinal, or general anesthetic). Exclusion criteria are lack of consent, multiple pregnancy, history of eclampsia or cardiovascular complications and contraindications to TXA (e.g. allergy, history of thromboembolism). A complete list of inclusion and exclusion criteria are detailed in Appendix 1. The division head of our high-risk maternal fetal medicine program (author JB) consulted with all obstetricians at our institution (21 total) and received support for participation. Because there is no centralized antenatal clinic, the study was advertised in the common areas of multiple clinics across our institution. Trained research assistants identified and approached eligible patients in several settings including routine antenatal visits with obstetricians (> 30 weeks gestational age), screening new admissions to the labour and delivery ward, and screening the booked elective CD list after receiving confirmation from a member of the clinical team that the patient was willing to speak with a research assistant about possible participation in research activity. Investigators were available to discuss specific concerns from participants if not addressed satisfactorily by research personnel. Upon receiving written, informed consent, final eligibility was confirmed after review by one of the physician investigators (AA, JB, JC, SC).

The funding agency (Academic Health Sciences Centre Innovation Fund—Ontario Innovation Fund Provincial Oversight Committee) had no role in the design, conduct, or analysis of the study. This manuscript adheres to the applicable CONSORT guidelines.

### Randomization, blinding, and allocation concealment

Patients providing informed consent and meeting all eligibility criteria were randomly assigned via computer-generated sequence (1:1), stratified by planned delivery modality (SVD or CD) at the time of consent. The randomization sequence was created and maintained by Clinical Trials Services at SHSC to maintain blinding and allocation concealment. Consenting participants were randomized (next kit in sequence issued by research pharmacy) when admitted to the labour and delivery unit. The research pharmacy at SHSC created identical appearing study kits, with unique codes based on the randomization sequence. The study kit was prepackaged to include a filled 10 mL syringe of IP, a 50 mL bag of 0.9% saline, and a secondary infusion line. The master list linking codes with group allocation was kept in the possession of the research pharmacy to maintain blinding until data collection and analysis were complete.

### Assigned interventions

Participants were randomized to one of two study groups:

1. TXA (Intervention group): TXA 1 g (10 mL syringe) diluted into 0.9% saline (50 mL).
2. Placebo (Control group): 0.9% saline (10 mL syringe) diluted into 0.9% saline (50 mL).

Immediately prior to use, one of the four physician investigators (AA, JC, JB, SC) prepared the infusion by injecting the 10 mL syringe of IP into the mini-bag and initiating the infusion. The intervention was infused though a free-flowing intravenous line over approximately 15 min. For participants having SVD, the infusion was initiated at the time of shoulder delivery. For participants having CD, the infusion was initiated during surgical site skin preparation.

All other obstetric and anesthetic care was at the discretion of the clinical team on the labour and delivery unit including diagnosis of PPH and administration of uterotonics beyond standardized protocols for oxytocin after delivery.

### Outcomes

#### Primary

The primary outcome of this pilot trial was to assess feasibility of delivering the study intervention with success defined as > 85% of randomised participants receiving the intervention.

#### Secondary

Among secondary outcomes, two were considered feasibility outcomes including time and cost to recruit the intended sample size (58 participants). The goal was to recruit the sample size in approximately 4 months. Other secondary outcomes are those related to the full trial including PPH (defined as blood loss greater 500 mL following SVD or greater than 1000 mL following CD), severe PPH (PPH requiring blood transfusion within 48 h of delivery, emergency hysterectomy or other operative intervention, admission to ICU or diagnosis of disseminated intravascular coagulation (DIC) within 24 h of delivery, and hospital length of stay. Safety related secondary outcomes, assessed during admission, 6, and 12 weeks post-delivery, including neonatal outcomes are detailed in Appendix 2.

### Sample size calculation

With a primary feasibility outcome of 85% of randomized participants receiving IP, 58 participants were required to achieve reasonable precision in the estimate of the primary outcome (95% CI 73.2–93%). Assuming approximately 4000 yearly deliveries at SHSC and an estimated recruitment rate of 5%, we estimated that this number of participants could be recruited in approximately 4 months. If feasible within the allotted time frame, this would have allowed for recruitment of 3408 patients, providing 80% power to detect a 35% relative risk reduction in PPH (6.1–4%), over approximately 2 years in a full, multi-centre trial, including affiliated centres from the Greater Toronto Area Obstetrical (GTA-OBS) Research Network (~ 65,000 deliveries/year).

### Statistical analysis

Demographic, feasibility, safety, and clinical endpoints are summarized with appropriate measures of central tendency and dispersion. Categorical outcomes are presented as frequency and percent. Inferential analysis is presented to identify gross differences between groups, recognizing that a pilot trial is underpowered for PPH incidence and thus only exploratory in nature. Continuous secondary feasibility and clinical outcomes were analyzed using a t-test continuous outcomes and chi-square (χ^2^) or Fisher’s Exact test for low cell counts. All available data was analyzed on an intention-to-treat principle using SAS Version 9.4 (SAS Institute, Cary, NC, USA). Given that clinical and safety outcomes were exploratory in nature, no adjustment was made for multiple comparisons.

### Ethics approval and consent to participate

REB Approval: Sunnybrook Health Sciences Centre # 418-2016, Health Canada Approval: Control# 207130.

## Results

**Figure 1:**
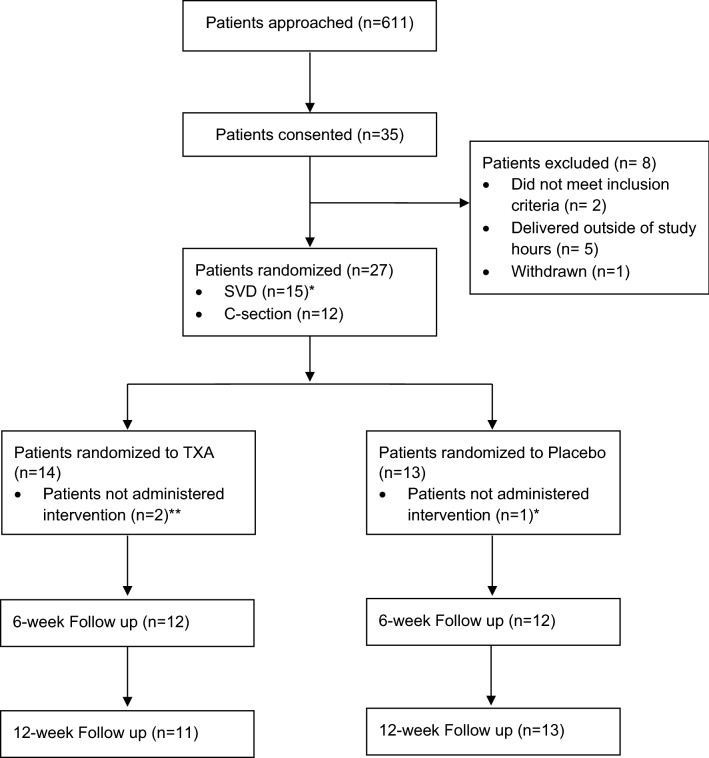
TAPPH-1 CONSORT flow diagram. *One participant for planned SVD underwent emergent C-Section randomized to placebo, did not receive intervention and did not receive TXA otherwise after review of records. **Participants delivered before intervention could be administered.

The CONSORT flow diagram details the number of participants approached, provided consent, were randomized, and received the allocated intervention (Fig. 1). Recruitment occurred from March 6th, 2018, to November 8th, 2018. The study period was extended due to low recruitment. During recruitment, 35 of 611 approached patients (5.7%) consented to participant in this study. Among these, five were not randomized because they were admitted outside of study hours (Monday to Friday, 07h00–23h00) where research personnel and investigators were not available to prepare and administer the intervention. Two did not meet inclusion criteria, one withdrew, and 27 were randomized. Thus, 4.4% of approached patients were ultimately randomized to receive either TXA or placebo. Among the 27 randomized, 24 (89%) received IP (3 patients delivered spontaneously before IP could be administered) (Fig. 1). Demographic characteristics are detailed in Table 1.

**Table 1:** Demographic Data.

	Total (n = 27)	TXA (n = 14)	Placebo (n = 13)
Age (years)	34.6 (3.8)	34.9 (3.6)	34.3 (4.1)
Height (cm)	164.6 (5.5)	163.3 (4.7)	165.9 (6.1)
Weight (kg)	89.8 (17.1)	92.3 (21.7)	87.3 (11.0)
BMI (kg m^−2^)	33.1 (6.3)	34.5 (8.0)	31.8 (3.8)
Previous childbirth			
0	13 {56}	4 {29}	9 {69}
1	9 {26}	7 {50}	2 {15}
2	3 {11}	2 {14}	1 {8}
3	2 {7}	1 {7}	1 {8}
Delivery method			
SVD	15 {56}	8 {57}	7 {54}
C-Section	12 {44}	6 {43}	6 {46}
Forceps assisted*			
Yes	0	0	0
No	15 {100}	8 {100}	7 {100}
Anesthetic modality			
Epidural	15 {56}	10 {71}	5 {38}
Spinal**	6 {22}	2 {14}	4 {31}
CSE**	5 {18}	1 {7}	4 {31}
GA**	1 {4}	1 {7}	0

Data presented as mean (SD) or count {%}.

*BMI* body mass index, *CSE* combined spinal epidural, *GA* general anesthesia, *SVD* spontaneous vaginal delivery, *TXA* tranexamic acid.

*Only for SVD.

**Only for C-Section.

Despite meeting the primary feasibility target (IP > 85%), participant accrual was significantly slower than expected despite extending the recruitment period. Over the 8-months, only 27 were randomized (~ 40 annually). This is 23.3% of the desired recruitment of 58 over 4 months (~ 174 annually). With approximately 4000 deliveries yearly at SHSC, this represents a very low recruitment rate of 1%. Extrapolating this rate to the full involvement of the GTA– OBS Research Network (~ 65,000 deliveries yearly), it would take approximately 5–6 years to achieve the sample size of a fully powered trial.

For clinical and safety secondary outcomes (Table 2), a single participant randomized to the placebo group experienced clinically diagnosed PPH. No other major clinical events related to PPH, or safety related outcomes associated (i.e. thromboembolic events) with TXA were observed. Four participants (3 in the TXA group and 1 in the placebo group) experienced minor adverse events including nausea, vomiting and dizziness. There were no neonatal adverse events recorded.

**Table 2:** Clinical and Safety Outcomes.

	Total (n = 27)	TXA (n = 14)	Placebo (n = 13)
PPH	1 {4}	0	1 {8}
Methylergonovine or prostaglandin F2 $\alpha$	0	0	0
Blood products administered	0	0	0
TXA outside of study protocol	1 {4}	0	1 {4}
Thrombotic complications	0	0	0
Acute kidney injury	0	0	0
Seizure	0	0	0
Intensive care unit admission	0	0	0
Neonatal complications	0	0	0
Nausea	4 {15}	3 {21}	1 {8}
Vomiting	2 {7}	2 {14}	0
Dizziness	1 {8}	0	1 {8}

Data presented as count {%}.

*PPH* postpartum hemorrhage, *TXA* tranexamic acid.

## Discussion

Our pilot trial exceeded the primary feasibility target of > 85% or randomized patients receiving IP (89%). Three randomized patients (1 TXA, 2 placebo) did not receive IP as they delivered spontaneously before the IP could be administered. In contrast, previous studies administering TXA at the same timepoint for vaginal delivery (delivery of the anterior shoulder), did not report any protocol deviations of randomized patients not receiving TXA^18,19^. However, it is unclear how rapid deliveries were addressed in these studies, i.e. if IP was administered following delivery.

Recruitment for randomized controlled trials is frequently challenging, particularly for those involving pregnant women, who have unique concerns regarding participation^20,21^. The vast majority of studies using TXA for the prevention of PPH have been conducted in low- or medium HDI countries, where recruitment situations may not reflect those observed in North America. One major exception to this rule is the TRAAP trial, published after our pilot trial had started, which recruited 4079 patients in France over a two-year period between January 2015 and December 2016, with a recruitment rate of ~ 13.6% of eligible deliveries^22^. The reasons for the significantly higher recruitment rate in the TRAAP study remain unclear but several factors may be considered.

Eligibility criteria can seemingly be eliminated as a contributing factor, as our criteria were more inclusive than previous studies (e.g. not requiring a cephalic presentation) attempting to maximize generalizability. One potential factor is resource and logistical limitations as restricting enrolment to patients delivering between 07h00 and 23h00, resulted in 5/35 (14%) of consented patients not being randomized. Around the clock availability of research personnel is cost prohibitive (~ $100,000 per year) and beyond the available resources for TAPPH-1. However, even taking these factors into account, the number of eligible patients who were approached, but did not consent was particularly high (94%). A likely explanation for this is differences in the recruitment reality, including differences in research culture or regulatory burden, observed in Canada or North America as a whole, compared to Europe or non-high HDI countries. A follow-up study to further explore the reasons for recruitment challenges is being considered.

The TRAAP study concluded that the relative risk of provider-assessed clinically significant PPH was lower in the TXA group although the primary outcome of PPH (determined quantitatively) was not significant (p = 0.07). The TRAAP trial also reported no significant difference in serious adverse events, including thromboembolism, seizure, or re-admission to hospital, although rates of nausea, vomiting and dizziness were higher with TXA. Notably, the TRAAP study only included patients delivering vaginally, limiting its generalizability, and necessitating further trials to address the large population of women who undergo CD. The follow-up study (TRAAP2) published in April 2021 by the same group randomizing 4551 women undergoing CD identified a relative risk of PPH of 0.84 (95% CI 0.75–0.94, p = 0.003) in those administered TXA^23^. An additional American study (NCT03364491) aiming to recruit 11,000 CD patients from 12 institutions has also been underway since March 2018 and is yet to be completed.

Despite apparent similarities in risk factors and incidence of PPH between Canada and France^24^, the TRAAP studies may not accurately reflect recruitment rates (136 yearly per participating institution) and patient behaviour in a North American setting. It is unclear from the CONSORT flow charts the number of patients approached to achieve the sample size and if the recruitment rates were significantly higher than that demonstrated herein. Further impeding feasibility in the Canadian context is the regulatory environment, where TXA for PPH requires a Health Canada Clinical Trial Application approval for off-label use. In Canada, a trial that requires a Health Canada Clinical Trial Application mandates any individual handling or administering the intervention completes specific training above and beyond standard Good Clinical Practices training (GCP). The training modules, such as Health Canada Division 5 (https://n2canada.ca/courses) require one to two full days of online training. This burden means that absent an institutional requirement for all nursing staff and physicians to be trained to handle investigational products, only the investigators can administer study interventions. In Canada, commercially available and approved medications, when repurposed or used in an off-label context, regardless of established clinical use (e.g., TXA in trauma, arthroplasty, or cardiac surgery) are subject to a regulatory regime identical to a newly developed drug. This includes specific documentation, accountability, and monitoring at each step. This significantly reduces the feasibility of a trial such as TAPPH-1 in Canada as this requires an investigator to be always readily available to administer the investigational product. This requirement did not appear to be necessary for the TRAAP studies in France. Both likely significantly contributed to the feasibility in France and the lack of feasibility in Canada. The implications of the regulatory environment in Canada are such that any trials aimed at repurposing existing medications for an off-label use in Canadian obstetrics, regardless of historical safety data, will face significant impediments and inhibit research. Alternative trial designs (e.g. cluster randomization by centre or deferred/no consent) are not feasible either. In an elective, non-emergent situation, where participants are potentially randomized to an off-label intervention, trial design cannot override the requirements for individual participant consent and credentials for administering the investigational product. Regarding trials with cluster randomization being employed, the B-free trial (NCT03928236) randomizes centres to two arms that are both considered standard of care and a no-consent model is ethically acceptable. Prophylactic administration of TXA is not yet standard of care. Neither is it an emergency (patients awake, not in extremis) therefore does not meet the threshold where obtaining prior informed consent is not practical or life threatening.

The generalizability of this trial is limited by its single centre nature. The regulatory environment within Canada is such that the specific Health Canada requirements for off-label trials would be applicable to any centre within Canada. Our institution is also a high-risk obstetric centre, and the study sample was recruited from a population with a higher prevalence of medical or pregnancy related issues and as such may also limit generalizability and our estimation of feasibility. Furthermore, because of a lack of 24-h coverage, our study also would not be applicable for emergency cases.

## Conclusions

We have presented here the feasibility data for the TAPPH-1 pilot trial, the first to assess the recruitment rates in a Canadian PPH prevention study. While there was an insufficient number of participants to properly address clinical and safety outcomes, our results are largely consistent with previously reported data. Furthermore, the value of this work is that publication of pilot feasibility trials, regardless of outcome, have been demonstrated to reduce waste in research and are critical for completion of randomized controlled trials^25^. Despite meeting the primary feasibility target of 85% of randomized patients receiving IP, we observed a much lower recruitment rate than expected, and therefore the full trial was determined not to be feasible. While several factors may have contributed to the low recruitment rate in this pilot study, we believe that cultural and regulatory differences specific to North America played a role, highlighting the importance of basing study feasibility on data specific to a given recruiting locale.

## Supplementary Information

Supplementary Information 1.

Supplementary Information 2.

## Data Availability

The datasets used and/or analysed during the current study are available from the corresponding author on reasonable request.

## Abbreviations

CD: Caesarean delivery
FDA: Food and drug administration
GCP: Good clinical practices
GTA-OBS: Greater Toronto area obstetrical research network
HDI: Human development index
IP: Investigational product
PPH: Postpartum haemorrhage
REB: Research ethics board
SHSC: Sunnybrook health sciences centre
SPIRIT: Standard protocol items: recommendations for interventional trials
SVD: Spontaneous vaginal delivery
TXA: Tranexamic acid
