# Supplementary material for: Tranexamic acid for the prevention of postpartum haemorrhage: the TAPPH-1 pilot randomized trial and lessons learned for trials in Canadian obstetrics
Source: Sci Rep. 2023 Mar 18;13:4512. doi: 10.1038/s41598-023-30947-8 (PMC10024764; doi:10.1038/s41598-023-30947-8)
Supplement: Supplementary file 1 — Supplementary Information 1. [file 41598_2023_30947_MOESM1_ESM.docx]

**Appendix 1 – Inclusion and exclusion criteria**

**Inclusion Criteria:**

- Confirmed singleton pregnancy
- Gestational age > 32 weeks
- > 18 years old

**Exclusion Criteria:**

- Lack of consent
- Eclampsia or pre-eclampsia in current pregnancy
- Imminent delivery (<2 hours) from presentation (ie. unable to complete informed consent, confirm eligibility, and obtain study kit from research pharmacy)
- Contra-indication to Tranexamic acid
  - Allergy to TXA
  - Color blindness
  - Active/history of venous or arterial thromboembolism
- History of hematuria
- Prothrombotic disease (Protein C or Factor V Leiden deficiency)
- Coronary artery disease
- History of congenital heart disease
- Peripheral arterial disease
- Unstable Arrythmia
- History of seizure disorder
- Renal failure
- Inability to comply with follow-up
