# Supplementary material for: Tranexamic acid for the prevention of postpartum haemorrhage: the TAPPH-1 pilot randomized trial and lessons learned for trials in Canadian obstetrics
Source: Sci Rep. 2023 Mar 18;13:4512. doi: 10.1038/s41598-023-30947-8 (PMC10024764; doi:10.1038/s41598-023-30947-8)
Supplement: Supplementary file 2 — Supplementary Information 2. [file 41598_2023_30947_MOESM2_ESM.docx]

**Appendix 2 – Safety related secondary outcomes**

- Thromboembolic complications
  - Deep venous thrombosis
  - Pulmonary embolism
  - Venous sinus thrombosis
- Seizure within 24h of delivery
- New onset renal failure during admission
- Health status of neonate at 6 and 12 weeks
- Minor adverse events known to be associated with TXA (nausea, vomiting, diarrhea, dizziness, headache, visual disturbances/changes, light-headedness)
